# Mesenchymal Migration on Adhesive–Nonadhesive Alternate Surfaces in Macrophages

**DOI:** 10.1002/advs.202301337

**Published:** 2023-05-21

**Authors:** Fulin Xing, Hao Dong, Jianyu Yang, Chunhui Fan, Mengdi Hou, Ping Zhang, Fen Hu, Jun Zhou, Liangyi Chen, Leiting Pan, Jingjun Xu

**Affiliations:** ^1^ The Key Laboratory of Weak‐Light Nonlinear Photonics of Education Ministry School of Physics and TEDA Institute of Applied Physics Nankai University Tianjin 300071 China; ^2^ State Key Laboratory of Medicinal Chemical Biology Frontiers Science Center for Cell Responses, College of Life Sciences Nankai University Tianjin 300071 China; ^3^ State Key Laboratory of Membrane Biology, Institute of Molecular Medicine National Biomedical Imaging Center, Center for Life Sciences School of Future Technology Peking University Beijing 100871 China; ^4^ Shenzhen Research Institute of Nankai University Shenzhen Guangdong 518083 China

**Keywords:** macrophage, mesenchymal migration, myosin IIA, nonadhesive surface, podosome

## Abstract

Mesenchymal migration usually happens on adhesive substrates, while cells adopt amoeboid migration on low/nonadhesive surfaces. Protein‐repelling reagents, e.g., poly(ethylene) glycol (PEG), are routinely employed to resist cell adhering and migrating. Contrary to these perceptions, this work discovers a unique locomotion of macrophages on adhesive–nonadhesive alternate substrates in vitro that they can overcome nonadhesive PEG gaps to reach adhesive regions in the mesenchymal mode. Adhering to extracellular matrix regions is a prerequisite for macrophages to perform further locomotion on the PEG regions. Podosomes are found highly enriched on the PEG region in macrophages and support their migration across the nonadhesive regions. Increasing podosome density through myosin IIA inhibition facilitates cell motility on adhesive–nonadhesive alternate substrates. Moreover, a developed cellular Potts model reproduces this mesenchymal migration. These findings together uncover a new migratory behavior on adhesive–nonadhesive alternate substrates in macrophages.

## Graphical abstract

Macrophages exhibit an exceptional mobility that they adopt a counterintuitive mesenchymal mode to migrate across the nonadhesive region on adhesive–nonadhesive alternate surfaces, which is insurmountable for other cell types. This motility is supported by podosomes formed on nonadhesive regions and can be enhanced by myosin IIA inhibition. This finding fills the void that mesenchymal migration can happen on nonadhesive surfaces.

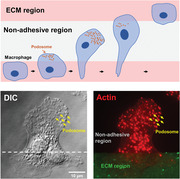

## 1 Introduction

Single‐cell migration involves two distinct modes, known as the adhesion‐dependent mesenchymal mode, and adhesion‐independent but contraction‐driven amoeboid mode.^[^ ^1^, ^2^ ^]^ Cells in mesenchymal mode typically exhibit a polarized morphology with a well‐defined leading‐edge and a uropod at the back.^[^ ^3^ ^]^ They generate force on substrates to facilitate migration via adhesion structures, e.g., focal adhesions (FAs)^[^ ^4^ ^]^ and podosomes.^[^ ^5^ ^]^ Quite differently, cells in amoeboid mode usually adopt irregular morphology with intracellular activity of actomyosin system to exert the contraction force on low/nonadhesive surfaces without adhesion‐associated structures.^[^ ^6^ ^]^ It is interconvertible between mesenchymal migration and amoeboid migration. Recently, it was reported that mesenchymal‐amoeboid transition could happen in low adhesive environment with strong physical confinement,^[^ ^7^ ^]^ which was proposed as a mechanism for cancer cells to adapt their migration mode to their environment. However, it is still unknown whether cells can adopt a mesenchymal morphology on nonadhesive surfaces.

Macrophages, serving as the first line of defense against exogenous pathogen, play crucial roles in various physiological and pathological processes based on their strong mobility.^[^ ^8^, ^9^ ^]^ Macrophages usually displayed a classic mesenchymal mode of migration in heterogeneous interstitial tissues in vivo,^[^ ^10^, ^11^ ^]^ with a relatively low speed compared to fast amoeboid cells, e.g., neutrophils. Macrophages also exhibit plasticity in morphology and migration mode, enabling them to deal with complex substrates, such as amoeboid migration on soft substrates or 3D environments and mesenchymal migration on stiff substrates.^[^ ^12^, ^13^, ^14^ ^]^ Extensive research has focused on the migration properties of macrophages on homogeneous substrates, but their mobility on heterogeneous substrates, which more closely resembles complex environments in vivo, still remains to be determined.

In the present work, using well‐established cell‐patterning technique to precisely control the available adhesive area, we investigate the migration of macrophages in heterogeneous substrates with alternate nonadhesive gaps. We observe an outstanding mobility that they adopt a counterintuitive mesenchymal morphology to cross the nonadhesive surfaces, which fills the void that mesenchymal migration can happen on nonadhesive surfaces.

## 2 Results

### 2.1 Mesenchymal Migration Can Happen on Adhesive–Nonadhesive Alternate Surfaces in Macrophages

**Figure 1:**
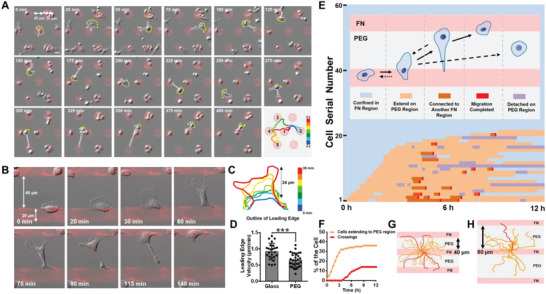
Mesenchymal migration can happen on the adhesive–nonadhesive alternate substrates in macrophages. A) A macrophage migrates from one adhesive FN island (diameter = 35 µm) to another island (gap distance = 40 µm) five times in 6 h. Color indicates different time points. Scale bar, 20 µm. B) Mesenchymal migration across a striped FN‐PEG pattern. The FN stripe is 20 µm in width with a gap distance of 40 µm. C) Outline of the macrophage leading edge in (B). Color indicates different time points. D) Statistical velocity of the leading edge on the glass and PEG surfaces. *N* = 27 for each group. Statistics were performed by Student's *t*‐test. Asterisks indicate statistical significance compared with the glass group, ***P < 0.001. E) Statistical schematic of macrophage migration on the adhesive–nonadhesive alternate surface. Watery blue, yellow, orange, red, and gray represented migration states, including cells being confined in the FN region, extending to the PEG region, being elongated and connecting to another FN region, a crossing completed, and being detached on the PEG region, respectively. F) Quantification of the fraction of cells extending to PEG and crossings in (E) over time. Migration trajectory of cells on striped patterns with gap distances of 40 µm G) and 80 µm H). Red traces represent completed crossings. Data was obtained from Movies S1, part SII (Supporting Information).

To fabricate a heterogeneous substrate, we used an adhesive–nonadhesive alternate surface composed of poly‐l‐lysine‐g‐PEG (pLL‐PEG, abbreviated as PEG in the following sections) and fibronectin (FN) extracellular matrix (ECM) with stripe and triangular lattice circular patterns. Protein‐repelling regents, such as PEG, prevent cell adhesion via resisting nonspecific protein adsorption.^[^ ^15^, ^16^ ^]^ Challenging this popular perception, we found that macrophages could actively migrate across the nonadhesive PEG region (**Figure** **1**A; Movie S1, part SI, Supporting Information). The cells exhibited no directional migration, but random locomotion, as visualized by the irregularity of the migration trajectory (Figure 1A). Unexpectedly, macrophages migrated over the nonadhesive region in a classical mesenchymal mode, other than an amoeboid morphology, with a well‐defined lamellipodia leading edge, an obvious uropod at the back and a typical four‐step migration behavior (Figure 1B; Movie S1, part SII, Supporting Information).^[^ ^17^ ^]^ In detail, the cell first extended out of the FN region with a subsequent elongation of the cell body. Afterward, the leading edge reached another FN region, followed by the uropod dissociation and contraction of the cell body (Figure 1B; Movie S1, part SII, Supporting Information). The forward velocity of the leading edge on PEG surface was 0.68 ± 0.04 µm min^−1^, which was lower than that on the glass surface (0.92 ± 0.05 µm min^−1^) (Figure 1C,D), indicating that PEG surface was not conducive for cell locomotion. We then observed some typical mesenchymal‐shaped cells, such as NIH 3T3 fibroblasts and MDCK epithelial cells on uniform substrates. They formed lamellipodia at the leading edges, with uropod at the rear, exhibited a four‐step migration behavior (Figure S1A,B, Supporting Information), as well as a relatively low migrating speed (≈1 µm min^−1^; Figure S1C, Supporting Information), demonstrating the mesenchymal nature of macrophage migration on the adhesive–nonadhesive alternate surface.

To quantitatively describe the statistical behavior of macrophages, we employed different colors to define different migration states of macrophages on adhesive–nonadhesive alternate substrates. That is, watery blue, yellow, orange, red and violet represented states of cells being confined in the FN region, extending on the PEG region, being elongated and connecting to another FN region, migration across‐PEG region completed (we styled that as “a crossing”) and being detached on the PEG region, respectively. Each horizontal line represented one single cell, while different colors at different time points indicated the transition of migration states (Figure S1E, Supporting Information). The selected view contained 60 cells, and the time‐lapse imaging lasted 12 h (Movie S1, part SIII, Supporting Information). All cells in the view were lined up according to the time order of extending to the PEG region. It was shown in Figure 1F that the ratio of cells that were able to extend to the PEG region peaked in 3 h at ≈36%, and that ≈20% of the cell could complete at least one crossing. 10 independent experiments were analyzed and obtained nearly the same results. The trajectory of cells in Movie S1, part SIII (Supporting Information), indicated random locomotion (only the first crossing was recorded) (Figure 1G). Varying the gap distance (from 40 to 100 µm), we found a maximal PEG gap distance of ≈80 µm that macrophage could make a crossing (Figure 1H; Movie S1, part SIV, Supporting Information). 3 independent experiments were performed with similar results.

We defined this locomotion by two abilities, namely, the ability to extend the cell body to the nonadhesive PEG region and the ability to elongate the cell body for reaching another adhesive region. Then, we observed the migration of 25 types of cells including cancer cells, cell lines, or primary cells on adhesive–nonadhesive alternate substrates. Almost none of the examined cell types own the ability to migrate across the adhesive–nonadhesive alternate substrates (Movie S2, parts SI–SIII, Supporting Information), which also confirmed the nonadhesive and homogeneous property of the PEG regions. Human neutrophil was observed to protrude out of the FN region in an amoeboid phenotype but could not elongate the cell body to reach another FN region. A few Raw264.7 cells, a mouse macrophage cell line, were observed to extend onto the PEG region (Movie S2, part SIV, Supporting Information). Only mouse and rat peritoneal macrophage (Movie S2, part SIV, Supporting Information) had a complete ability to migrate across the nonadhesive PEG region. Thus, we suggest that this motility on adhesive–nonadhesive alternate substrates is a unique ability for macrophages.

To test whether this cell motility was PEG‐dependent or FN‐dependent, we replaced PEG and FN with another cell‐repelling regent, F127 as well as another type of ECM, collagen I. It showed that macrophages could also migrate across all these adhesive–nonadhesive alternate substrates, proving the universality of this motility on alternate nonadhesive surfaces (Movie S2, part SIV, Supporting Information).

To characterize the motility of macrophages, we first cultured cells on a pure PEG surface. Interestingly, we found that they kept a nonadherent round state (Figure S2A and Movie S3, part SI, Supporting Information). By contrast, when the cell touched the rim of the FN region, it spread rapidly on this adhesive region (Figure S2B and Movie S3, part SI, Supporting Information). Further, we observed that adhered cells elongated cell body to the PEG region and retracted (Figure S2C and Movie S3, part SII, Supporting Information), or else they would lose adhesion on the FN region and subsequently turn round‐shaped on the PEG region (Figure S2D and Movie S3, part SII, Supporting Information), suggesting that macrophage motility needed a preadhesion on the FN region. The average time between detaching from FN region and turning round was 11.9 ± 5.8 min (*N* = 20). These results showed that cell could not maintain spreading morphology when losing connection with the FN region, even this connection was a small fraction of the cell body. This prerequisite of FN adhesion, we guess, may be associated with integrin‐based adhesion signaling pathway.

### 2.2 Podosomes Enriched on nonadhesive Regions of the adhesive–Nonadhesive Alternate Substrates

It is widely accepted that cells require adhesion to resist liquid fluctuation and internal Brownian movement.^[^ ^18^, ^19^ ^]^ Thus, macrophages are likely to form adhesion on the PEG region, rather than suspend on it. Scanning electron microscopy suggested that macrophages adhered tightly to the PEG surface without interspaces (Figure S3A, Supporting Information). Also, gentle mechanical stimulations (by a glass electrode mounted on a three‐axis hanging joystick oil hydraulic micromanipulator) with the speed of ≈1 µm s^−1^ could not move the cell on the PEG region (Figure S3B and Movie S4, Supporting Information), suggesting that macrophages indeed adhered onto the nonadhesive PEG region.

**Figure 2:**
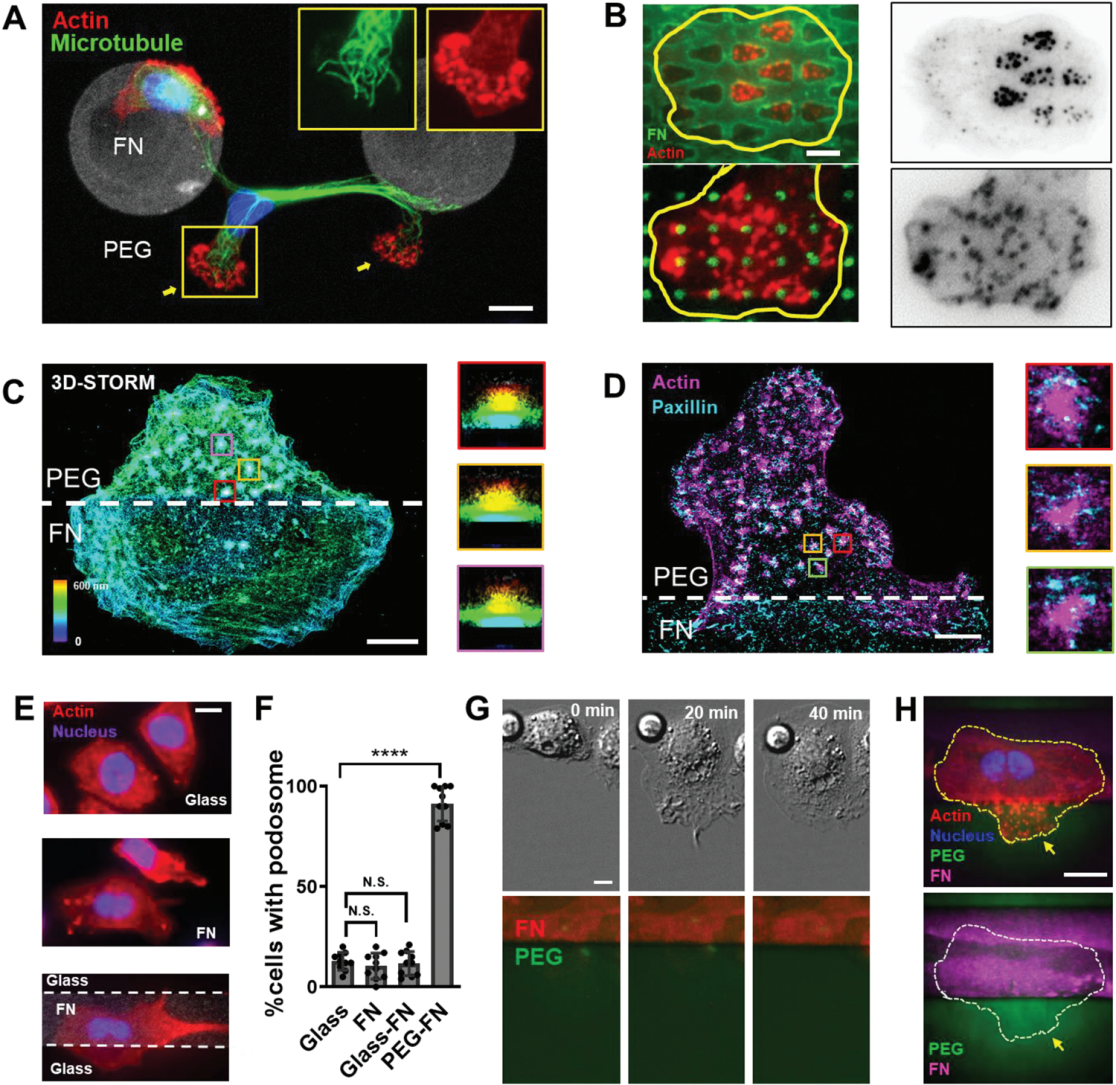
Podosomes form on nonadhesive part of the adhesive–nonadhesive alternate substrates. A) Immunostaining images of macrophage labeled with tubulin (green) and actin (actin). Podosomes are enriched on the PEG region. Scale bar, 10 µm. B) Macrophage on meshwork pattern and dots‐array pattern labeled by phalloidin. Podosomes avoid the FN regions. Scale bars, 10 µm. C) Representative 3D‐STORM image of F‐actin and the spatial organization of individual podosome. Scale bar, 5 µm. D) Cells form podosomes when extending to the PEG region (actin and paxillin labeled). Scale bar, 5 µm. E) Cells do not form podosomes on glass, FN, or glass‐FN surface, respectively. Scale bar, 10 µm. F) Quantification of the ratio of podosome‐positive cells cultured on glass, FN, FN‐glass, and PEG‐FN. Statistics were performed by Student's *t*‐test. Asterisks indicate statistical significance compared with the glass group, N.S., no significance, ***P < 0.001. *N* = 10 for each group. G) Macrophages do not degrade PEG (labeled by CF‐568) during migration. Scale bars, 5 µm. H) Podosomes in macrophages do not degrade PEG (labeled by CF‐568) during migration. Scale bars, 10 µm.

To investigate why macrophages could extend and adhere on the nonadhesive PEG region of the adhesive–nonadhesive alternate substrates, we first labeled F‐actin and *α*‐tubulin were in fixed cells. We thus found that F‐actin was highly enriched in the PEG regions as puncta, while microtubules distributed without region‐specificity (**Figure** **2**A). Using other micropatterns, including dot arrays and meshwork patterns, we further showed that F‐actin puncta were exclusively distributed on the PEG regions but avoided the FN regions (Figure 2B; Figure S3C, Supporting Information). Similar results were observed on the F127 nonadhesive surface (Figure S3D, Supporting Information) and on glass/HMDS/collagen adhesive surfaces (Figure S3E–G, Supporting Information) in mouse macrophages, suggesting that the formation of F‐actin puncta was not PEG/FN‐dependent. We identified these F‐actin puncta as podosomes through observations of the distinctive snow‐man‐like spatial organization^[^ ^20^, ^21^ ^]^ by 3D‐STORM^[^ ^22^ ^]^ (Figure 2C) and the ring (paxillin) +core (actin) distribution (Figure 2D). Furthermore, cells on glass, FN, and glass‐FN substrates showed no evident podosomes (Figure 2E). Statistical data showed that the percentages of podosome‐positive cells were 12.8 ± 1.4%, 10.8 ± 2.0%, 11.7 ± 1.9%, and 91.4 ± 2.8% on the glass, FN, glass‐FN and PEG‐FN substrates, respectively (Figure 2F). Together, these results demonstrated that the formation of podosomes depended on the adhesive–nonadhesive alternate substrates.

To exclude the possibility that macrophage degraded PEG and facilitate following migration, we used dye‐tagged PEG and found that no PEG was eliminated by macrophages (Figure 2G,H). At least 30 cells from five independent experiments were analyzed with similar results obtained. These observations indicated that podosomes adhered, but not degraded the PEG surfaces, and also demonstrated the mesenchymal migration mode of macrophage from the perspective of characteristic adhesion structures^[^ ^17^ ^]^ on the adhesive–nonadhesive alternate substrates.

### 2.3 Mesenchymal Migration on the adhesive–Nonadhesive Alternate Substrates Depends on Podosomes

**Figure 3:**
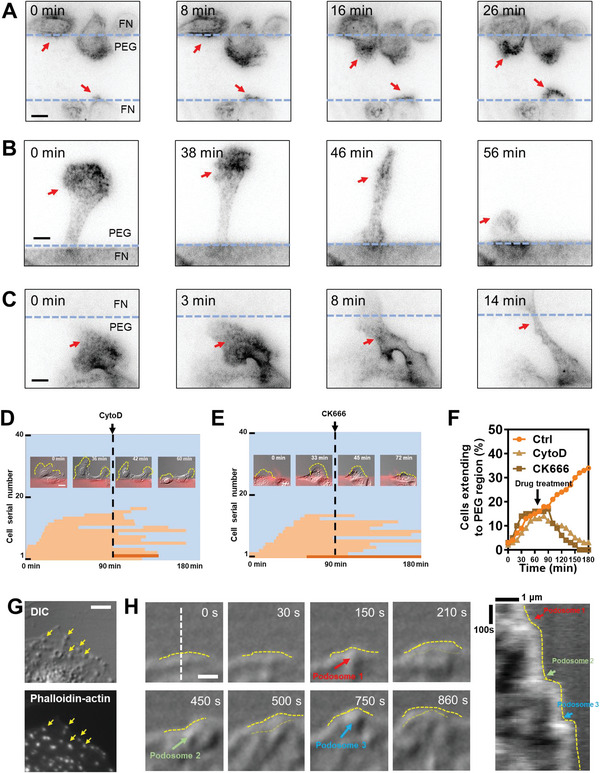
Podosomes support mesenchymal migration on the adhesive–nonadhesive alternate substrates. A) Cells form podosomes when extending to the PEG region (labeled by SiR‐actin). Scale bar, 5 µm. B) Podosomes disassemble before cells retracted back to the FN region. Scale bar, 5 µm. C) Podosomes disassemble when this part reaches the FN region. Scale bar, 5 µm. D,E) Migration diagram and time lapse‐imaging of CytoD D) and E) CK666 group. Scale bars, 20 µm. F) Fraction of cells extending to the PEG region over time in the CytoD and CK666 groups. G) Protuberances in DIC images corresponde with podosomes labeled by phalloidin. Scale bar, 5 µm. H) Membrane of the leading edge moves forward upon proximal podosomes appearing. Yellow dashed lines indicate the outline of leading‐edge membrane. The light dashed yellow lines (210, 500, and 860 s images) indicate the outline of the membrane leading edge in the last frame. Arrows indicate podosomes. Right panel: kymograph of the white dashed line. Arrows indicated podosomes formation on the leading edge of the cell. Scale bar, 1 µm.

To investigate the role of podosomes in the migration of macrophages on the adhesive–nonadhesive alternate substrates, we used SiR‐actin, a living‐cell F‐actin labeling probe to examine the dynamic of podosomes by TIRF microscopy. Podosomes clearly appeared at the front of the cell when it extended to the PEG region (**Figure** **3**A; Movie S5, part SI, Supporting Information) and disappeared before they retracted back to the FN region (Figure 3B; Movie S5, part SII, Supporting Information). When the cell reached another FN region, podosomes disassembled as well (Figure 3C; Movie S5, part SIII, Supporting Information). This observation indicated that podosomes were essential for the cell migration on adhesive–nonadhesive alternate substrates. At least 12 cells for each case were analyzed from three independent experiments with similar observations obtained. Inhibiting podosome formation by cytochalasin D (CytoD, an actin polymerization inhibitor) or CK666 (an Arp2/3 inhibitor) decreased macrophage motility on the alternative nonadhesive substrates, suggesting a supporting role of podosomes in this migration (Figure 3D–F; Figure S4A,B and Movie S6, Supporting Information). Additionally, we performed siRNA targeting WASP, a protein indispensable for podosome formation^[^ ^23^, ^24^ ^]^ to block the podosome formation in macrophages. The resulted showed that the treatment with WASP siRNA reduced the motility of macrophages on the adhesive–nonadhesive alternate surface (Figure S4C,D and Movie S7, part SII, Supporting Information), demonstrating the necessity of podosome for this motility. Three independent experiments were performed and similar results were obtained.

We also observed dynamic protuberances in the thin lamella layer by DIC imaging (Movie S8, Supporting Information). Fluorescence‐labeled F‐actin revealed that each protuberance in DIC images matched single podosomes (Figure 3G; Figure S5A, Supporting Information). Based on this observation, we analyzed the podosome dynamic during the process of cell spreading on PEG region. It was found that the area of cell body protruded on PEG region was proportional to the number of podosomes (Figure S5B,C, Supporting Information). Furthermore, it was observed that membrane protruded after the appearance of a single proximal podosome on the PEG region (Figure 3H; Movie S8, Supporting Information), suggesting that podosomes drove the actin polymerization and membrane protrusion on the PEG surfaces as a “piling” on the PEG surface. To quantify the relationship between membrane protrusion and podosome formation, we measured the normalized area change before and after the appearance of single membrane‐proximal podosomes. The maximum speed of membrane protrusion was observed ≈20 s after the formation of podosomes (Figure S5D,E, Supporting Information). These observations together demonstrated that podosomes supported the adhesion and motility of macrophages on adhesive–nonadhesive alternate substrates.

### 2.4 Inhibition of Myosin IIA Promotes Mesenchymal Migration on Adhesive–Nonadhesive Alternate Substrates

**Figure 4:**
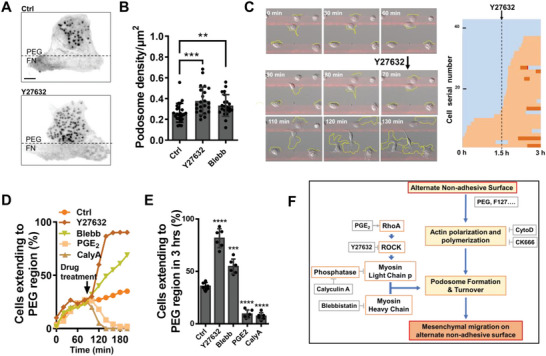
Inhibition of myosin IIA increases podosome density and promotes cell motility on the adhesive–nonadhesive alternate substrates. A) Representative images of macrophages with F‐actin labeled in control and Y27632 groups. Scale bar, 5 µm. B) Quantification of podosome density in the control, Y27632, and Blebb groups. *N* = 20 for each group. C) Y27632 promotes cell motility on the adhesive–nonadhesive alternate surface. PEG regions are 40 μm width and FN regions are 20 μm in width. D) Representative fraction of cells extending to the PEG region over time in the control, Y27632, Blebb, PGE_2,_ and CalyA groups. E) Quantification of the ratio of cells extending to the PEG region in different groups 1.5 h after the different drug treatments. *N* = 6 for each group. F) Schematic of regulatory pathway in macrophage migration on adhesive‐nonadhesive alternate substrates.

Considering the supporting role of podosome in this unique migration behavior, we reasoned that upregulation of podosome may facilitate cell motility on the adhesive–nonadhesive alternate substrates. Inhibition of myosin IIA, was reported to promote the podosome formation.^[^ ^25^, ^26^, ^27^ ^]^ We thus applied Y27632 (a ROCK inhibitor) and Blebbistatin (Blebb, a myosin II ATPase inhibitor). As expected, inhibition of myosin IIA increased the density of podosomes (**Figure** **4**A), which was 0.26 ± 0.01, 0.37 ± 0.03, and 0.33 ± 0.02 µm^−2^ for the control, Y27632, and Blebb groups (Figure 4B). Furthermore, cell motility on the adhesive–nonadhesive alternate substrates was enhanced greatly upon myosin IIA inhibition (Figure 4C; Figure S6A,B and Movie S9, part SI, Supporting Information). We also applied these drugs in macrophages on pure PEG surfaces and found that cells remained a round, nonadhering morphology (Figure S6C,D and Movie S9, part SII, Supporting Information), demonstrating the prerequisite of adherence on FN region.

By contrast, activating myosin IIA by prostaglandin E_2_ (PGE_2_, a Rho‐ROCK pathway activator)^[^ ^28^ ^]^ and calyculin A (CalyA, a phosphatase inhibitor)^[^ ^7^ ^]^ blocked the formation of podosomes (Figure S6F,H, Supporting Information) and reduced the macrophage motility (Figure 4D,E; Figure S6E,G and Movie S9, part SIII, Supporting Information). In detail, the ratio of cells extending to the PEG region within 3 h was 35.9 ± 1.9%, 81.7 ± 3.7%, 54.7 ± 2.9%, 7.2 ± 2.0%, and 6.1 ± 1.4% for the control, Y27632, Blebb, PGE_2_ and CalyA groups, respectively (Figure 4E). Therefore, inhibition of myosin IIA pathway promote podosome formation, and facilitate macrophage migration on the adhesive–nonadhesive alternate substrates (Figure 4F), suggesting the negative role of myosin IIA in this migratory behavior in macrophages.

### 2.5 A developed CPM Model for Mesenchymal Migration on Adhesive–Nonadhesive Alternate Substrates

**Figure 5:**
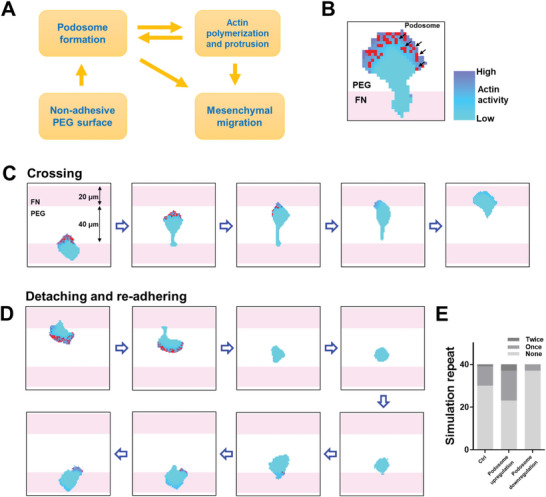
Theoretical simulation of cell migration on the adhesive–nonadhesive alternate substrates. A) Schematic of the relationship between podosome formation, actin polymerization, PEG and cell migration in the developed CPM model. B) CPM cell model. Color bar encodes the intensity of actin activity. Red pixels indicate the podosomes. C) Simulation presentation of a macrophage crossing a 40 µm PEG gap. D) Simulation presentation of cell detaching, turning round, and readhering on the adhesive–nonadhesive alternate surface. E) Number of crossings in 40 repeats of simulation in 20 µm FN stripe and 20 µm FN stripe with podosome upregulated.

The above intriguing observations of macrophages led us to develop a cellular Potts model (CPM)‐based theoretical framework to describe the motility of macrophages on the adhesive–nonadhesive alternate surface.^[^ ^29^ ^]^ Two fundamental assumptions, i.e., nonadhesive surfaces induced the formation of podosomes, and podosomes supported cell migration, was introduced into the model. The simulative cells possessed an intrinsic activity gradient of actin that led to migration.^[^ ^30^, ^31^ ^]^ Podosomes, which enable adhesion and locomotion on PEG, were induced by the PEG surface when cells reached the PEG surface. A positive feedback path was constructed that podosomes supported the actin polymerization and protrusion (Figure S5 and Movie S8, Supporting Information), while the activating of actin promoted the formation of podosomes (**Figure** **5**A,B). A negative pathway was set that podosomes disassembled when cells reached another FN region. Detailed equations and parameters were described in Materials and methods. The simulation results showed that actin polarization produced a leading edge of the cell with podosomes appearing when extending to the PEG region (Figure 5C; Movie S10, part SI, Supporting Information). When the uropod of the cell was detached from FN, the cell turned round on the PEG region in a dozen minutes (Figure 5D; Movie S10, part SII, Supporting Information). Afterward, the cell reached another FN region and readhered to it (Figure 5D; Movie S10, part SII, Supporting Information).

Considering the supporting role of cell motility on the adhesive–nonadhesive alternate surface (Figure 5A,B), we then upregulated and downregulated the density of podosome generation and resulted in a higher and lower probability of crossing as a consequence (Figure 5E; Movie S10, part SIII, Supporting Information). Moreover, mesenchymal migration in a triangular lattice pattern was also simulated in Movie S10, part SIV (Supporting Information). These simulation results were highly consistent with our experimental observations (Figures 1, 2, and 4A–C). Our model, on the other hand, can provide a powerful framework to describe cell locomotion on heterogeneous substrates.

## 3 Discussion

It is widely acknowledged that cells cannot migrate on nonadhesive substrates in mesenchymal mode lacking adhesion to generate pulling force. By contrary, they adopt amoeboid mode to migrate with cell–substrate friction from intracellular movement of actomyosin.^[^ ^32^ ^]^ Recently, it was reported that slow mesenchymal cells (e.g., NHDF cells) under physical confinement could convert to amoeboid migration on nonadhesive surfaces.^[^ ^7^ ^]^ In the present work, we found a disruptive migratory behavior that mesenchymal migration can happen on adhesive–nonadhesive alternate substrates in macrophages. The mesenchymal nature of macrophage migration on the adhesive–nonadhesive alternate substrates was verified through the cell morphology (Figure 1 A,B), a typical four‐step migration behavior (Figure 1B), migration velocity (Figure 1C,D; Figure S1, Supporting Information), as well as the characteristic adhesion structure of podosome (Figure 2A,B).^[^ ^17^ ^]^

Our observations, including SEM (Figure S3A, Supporting Information), mechanical stimulation (Figure S3B, Supporting Information), DIC imaging (Figure 2G,H), etc., demonstrated that macrophages adhered on PEG surfaces via podosomes, which was revealed by immunofluorescence (Figure 2A,B; Figure S3C, Supporting Information) and drug intervention (Figure 3D–F; Figure S4A,B, Supporting Information). This adhesion enabled the cell to generate pulling force for mesenchymal locomotion. As regards how podosomes adhere to PEG, it is still unclear and call for further investigations. We reasoned that some proteins in the podosomes might penetrate through the interspace between PEG chains and touched the covered adhesive substrates, as suggested in previous study.^[^ ^33^ ^]^ Overall, this powerful adhering ability of the podosomes in macrophages endows them with the mobility on the adhesive–nonadhesive alternate substrates.

Migration through the ECM gaps was mostly associated with the amoeboid mode.^[^ ^34^, ^35^, ^36^ ^]^ Recent studies also observed that some cells^[^ ^37^, ^38^ ^]^ could overcome short gaps of ≈10 µm with mesenchymal mode in vitro. Of particular note is that those c ells used protrusions to move across gaps much smaller than the cell size without extreme elongation of the cell body or adhesion on nonadhesive surfaces. Quite differently, in the present work macrophages were observed to migrate across nonadhesive PEG gaps (maximum 80 µm in Figure 1H) on the adhesive–nonadhesive alternate substrates in mesenchymal mode. As the first line of defense in innate immunity In vivo, macrophages need to perform potent migration through complex ECM gaps unsuitable for adhesion (e.g., collagen‐free zones caused by burn injury) in response to infection or injury.^[^ ^1^, ^36^, ^39^, ^40^ ^]^ This new‐found behavior of migration we unveiled endows macrophages with enormous adaptability to migrate through these complex interstitial tissues.

Our results showed an interesting phenomenon that cells needed preadhesion on the FN region for further locomotion (Figure S2A–D; Movie S3, Supporting Information). Inhibition of myosin IIA significantly enhanced cell motility on adhesive–nonadhesive alternate surfaces (Figure 4C–E; Movie S9, part SI, Supporting Information), while cells on pure PEG maintained round morphology after inhibiting myosin IIA (Figure S6C,D and Movie S9, Supporting Information), validating the necessity of preadhesion of this unique motility. In a previous study, cells tended to form podosome‐like adhesions on fluid surfaces lacking traction forces, whereas they habitually formed FAs on rigid surfaces,^[^ ^41^ ^]^ indicating that podosomes might form on substrates adverse to force generation. In the present work, macrophages form podosomes on nonadhesive regions, while they could form FAs on FN regions in the same cell (Figure 2D). This coexisting of FAs and podosomes in the same cell was also observed on uniform substrates in our prior study.^[^ ^21^ ^]^ Moreover, binding to ECM via integrin‐mediated FAs activated PI3K‐Akt pathway,^[^ ^42^ ^]^ which, as previously reported, promoted cytoskeleton rearrangement, e.g., podosome formation.^[^ ^43^, ^44^ ^]^ Therefore, we reasoned that activation of adhesion signaling on ECM was necessary for the formation of podosome on PEG regions in the same cell. These observations together indicated a whole‐cell coordination biomechanical network that preadhesion on ECM at the rear triggered podosome formation at the leading edge of macrophages on the adhesive–nonadhesive alternate substrates. Nevertheless, more detailed molecular mechanism needs to be addressed in the future.

Previous studies reported that podosomes selectively assembled on the adhesive region in micropatterned substrates. Specifically, peripheral blood mononuclear cells (PBMCs) derived dendritic cells,^[^ ^45^ ^]^ THP‐1^[^ ^46^ ^]^ and PBMCs^[^ ^47^ ^]^ derived macrophages were found to form podosomes on FN adhesive regions, opposite to our observations in primary peritoneal macrophages (Figure 2A,B; Figure S3C, Supporting Information). We reason that different types of cells and distinct culture conditions led to contradictory observations. For example, primary peritoneal macrophages formed podosomes immediately on our adhesive–nonadhesive alternate substrates after isolation, while on uniform substrates they needed 18 h in vitro culture to form enriched podosomes as reported in our previous study.^[^ ^21^ ^]^

Our simulation model, which was based on two assumptions, i.e., adhesive–nonadhesive alternate surface induced podosome formation and that podosomes supported migration (Figure 5A; Movie S10, Supporting Information), reproduced our experimental observations, confirming the validity of these assumptions from both experimental and theoretical perspectives (Movies S10 and S11, Supporting Information). In addition, considering the randomness and nonpreference nature of locomotion in macrophages (Figure 1G,H), we speculated that more cells would extend to PEG region on narrower FN stripes. As expected, the cell was found extending to the PEG region more easily on the 10 µm stripes than on 20 µm stripes with a higher frequency of crossings in the model (Figure S7A,B and Movie S11, part SI, Supporting Information). Subsequent experimental results showed that narrow stripes of 10 µm indeed enhanced the macrophage motility (Figure S7C,D and Movie S11, part SII, Supporting Information), validating the reliability and predictability of our model.

Taken together, our work uncovered a new migratory behavior on adhesive–nonadhesive alternate substrates and its podosome‐dependent regulatory mechanism, which gained new insight into the mobility of macrophage as the innate immune cells, as well as enrich our understanding of cell migration.

## 4 Experimental Section

### Ethics Statement

The animal protocol in this study conformed to the Guide for the Care and Use of Laboratory Animals (the Guide, NRC 2011), and it was also approved by the Institutional Animal Care and Use Committee at Nankai University (Approval ID 2021‐SYDWLL‐000072).

### Animals, Antibodies, and Drugs

C57BL/6 mice and rat were obtained from Beijing Vital River Laboratory Animal Technology Co., Ltd (China). Alexa Fluor 647‐conjugated phalloidin was from Invitrogen (A22287, USA). Primary antibodies of *α*‐tubulin and paxillin were from Abcam (ab7291 and ab32084, USA). Alexa Fluor 647‐conjugated secondary antibodies (Invitrogen) and CF568 succinimidyl ester (Biotium, USA)‐conjugated secondary antibodies were used for single‐color and two‐color STORM. The drugs including cytochalasin D, PGE_2_, blebbistatin, Y27632 were purchased from Sigma (C2618, P5640, 203392, Y0503, USA). Calyculin A (CalyA) was purchased from Glpbio (GC18106, USA). CK666 was purchased from Millipore (182515, USA)

### Cell Isolation and Culture

C57BL/6 mice were sacrificed strictly according to institutional guidelines. Phosphate buffer solution (PBS) (NaCl 150 × 10^−3^ m, KCl 5.4 × 10^−3^ m, CaCl_2_ 2 × 10^−3^ m, MgCl_2_ 1 × 10^−3^ m, glucose 10 × 10^−3^ m, and HEPES 10 × 10^−3^ m, pH = 7.4) was injected into the enterocoelia of mouse followed by gentle washing of minutes. The peritoneal cells were collected and isolated by centrifugation at 200*g* for 8 min. Then cells were resuspended in RPMI1640 medium (Gibco, USA) with 10% FBS in a humidified incubator with 5% CO_2_ at 37 °C following culture on micropatterned chips. The adherent cells containing approximately 95% macrophages determined by immunostaining for Iba‐1 were used in the following experiments.

### Fabrication of Micropatterned Substrate

Micropatterned substrates were prepared as previously described.^[^ ^48^ ^]^ In brief, glass substrates (*φ* = 25 mm) were immersed in chromic acid lotion for thorough cleaning and then dried at 80 °C for 20 min. Afterward, they were put into a vacuum desiccator containing hexamethyldisilazane (HMDS, Sigma, USA) for vapor phase deposition for at least 30 min. Then positive photoresist (RuiHong, China) was spun onto the HMDS‐coated substrates. Then, the substrate was exposed to UV light through a chrome‐based photomask with designed patterns. Exposed substrate was dissolved and removed by developing solution, subsequently. So far, glass substrates were fabricated. For following cell‐patterning, the substrates were treated with oxygen plasma treatment for 2 min to remove HMDS uncovered by photoresist. Afterward, the substrate was treated with acetone and rinsed with isopropanol and deionized (DI) water to dissolve the remained photoresist. Then, 0.1 mg mL^−1^ poly‐l‐lysine‐poly (ethylene glycol)−silane (pLL‐PEG) (SuSoS, Switzerland) solution was spread on the substrate for 30 min. Subsequently, 100 µg mL^−1^ fibronectin (FN, BD, USA) or collagen I (Macklin, China) was spread onto the prepared substrates for 30 min. After the FN/collagen solution was removed, prepared cell suspension was added on the substrates at 37 °C for 1 h to enable adhesion. After washing away the suspending cells, the resulting patterned substrates were placed in 37 °C and 5% CO2 for following experiments. For human neutrophil, cell suspension was added onto micropatterned substrates at room temperature to maintain the resting states.

For substrates fluorescence labeling, the FN/collagen I solution was mixed with a reactive dye (CF488A succinimidyl ester protein labeling kit, Biotium, USA) for 1 h at 7.4 pH. CF488‐FN/collagen I was excited by a mercury lamp using a 485/20 nm excitation filter, and fluorescence emission was collected through a 510 nm emission filter.

### Time‐Lapse Living Cell Imaging

Cells cultured on micropatterned substrates were placed in a CO_2_ incubator (INUB‐ZILCSGH‐F1, Tokai Hit, Japan) mounted on an inverted optical microscope (Ti‐E, Nikon, Japan) with an sCMOS (ORCA‐flash4.0, Hamamatsu, Japan). Images were captured in multichannel mode to obtain bright field data and fluorescence data at the same time. Obtained data was analyzed by ImageJ software.

### SiR‐Actin Staining

For labeling actin in living cells, they were incubated with 200 × 10^−9^ m SiR‐actin (CY‐SC002, Cytoskeleton, USA) with 1 × 10^−6^ m verapamil for 4 h in RPMI1640 medium. Then, cells were imaged without washing out probes at excitation beam of 647 nm from a TIRF objective mounted on Nikon TiE invert microscopy.

### Small RNA Interference (siRNA)

Cells were transfected using Lipofectamine3000 (Invitrogen) according to the manufacturer's instructions. Macrophages were cultured for 2 h in complete 1640 medium after being isolated from mouse enterocoelia. WASP siRNA was purchased from Genepharma (Shanghai, China). In every transfection, a ctrlRNA was used as control. Transfection efficiency was determined by fluorescence probe‐labeled RNA. Cells were transfected in WASP siRNA/ctrlRNA for 12 h before experiments.

### Immunofluorescence

Cells were fixed in 4% paraformaldehyde for 20 min followed by membrane permeability in blocking buffer (3% BSA and 0.05% Triton X‐100 in PBS) for 20 min. Then cells were stained with primary and secondary antibodies in blocking buffer for 1 h, respectively, or fluorescence‐probe‐labeled phalloidin for 1 h. Then, the sample was washed with PBS for the following imaging.

### 3D‐STORM Super‐Resolution Microscopy

3D‐STORM was performed as previously described.^[^ ^22^ ^]^ In brief, samples were mounted on glass slides using a standard STORM imaging buffer consisting of 5% (w/v) glucose, 100 × 10^−3^ m cysteamine, 0.8 mg mL^−1^ glucose oxidase, and 40 µg mL^−1^ catalase in Tris‐HCl (pH 7.5). A cylindrical lens was inserted into the imaging path to introduce astigmatism so that images of single molecules were elongated in opposite directions for molecules on the proximal and distal sides of the focal plane. Data was collected at 110 frames per second using an Andor iXon Ultra 897 EM‐CCD, for a total of ≈50 000 frames per image. 3D‐STORM raw data were processed according to previously described methods.^[^ ^22^ ^]^

### 3D‐STORM Data Analysis

To reconstruct the 3D distribution of different cortical proteins, the *x*, *y*, and *z* coordinates of all signal points in each selected region were extracted as TXT files. Using a custom‐written MATLAB routine, the *z*‐distribution profiles for each cortical protein were obtained from the projection of the graphics in the *z* direction and statistical analysis of the *z*‐coordinates of all signal particles. The *z*‐distribution profiles were fitted by a smooth curve using Origin software.

### Scanning Electron Microscopy and Atomic Force Microscopy

Cells were fixed in 4% glutaraldehyde overnight at 4 °C. Then, cells were washed in PBS in three times for 10 min. For dehydration, cells were immersed in 30%, 50%, 70%, 80%, and 90% ethanol on ice for 15 min, respectively. Afterward cells were treated with absolute ethyl alcohol on ice for 10 min three times. Dehydrated cells were placed in an air dry oven for complete dry. Before imaging, the sample was coated with gold for better conductivity. The imaging was carried out on an atomic force microscopy (SmartSPM, AIST, USA) or a scanning electron microscopy (SU3500, Hitachi, Japan).

### Statistical Analysis

All data are presented as mean ± standard error of mean (SEM) from at least three independent experiments. The statistical comparison between the two groups was carried out using unpaired Student's *t*‐test (GraphPad Prism 6), and the analysis for profiles of protein distribution in the *z*‐direction was using Origin software. Statistical significance was defined as *P < 0.05, **P < 0.01, ***P < 0.001, ****P < 0.0001; n.s., not significant (*P* > 0.05).

### Cellular Potts model

To simulate macrophage migration on adhesive–nonadhesive alternate surface, a developed cellular Potts model (CPM) was applied using the MATLAB software based on previously reported.^[^ ^29^, ^30^ ^]^ In a grid‐based plane, several connected grid sites defined a single cell, which moves randomly by copying their identity into neighboring grid in order to minimize a global energy function (Hamiltonian *H*). The probability of copying (*P*_copy_) depends on the energy variance (Δ*H*) before and after copying, the function takes the form of

(1) $$P_{\mathrm{copy}}\left(i\to j\right)=\left\{\begin{array}{c} 1,\;\Delta H>0\\ \exp(-\Delta H/T) \end{array}\right.$$

where *T* is the thermodynamic temperature, Δ*H* is the sum of variance of cell surface mechanics (CSM), substrate adhering mechanism, actin skeleton energy and anchoring energy

(2) $$\Delta H=\Delta H_{\mathrm{CSM}}+\Delta H_{\mathrm{substrate}}+\Delta H_{\mathrm{act}}+\Delta H_{\mathrm{anchor}}$$

When considering Δ*H*_CSM_ only, the function was shown below

(3) $$\begin{array}{ccc} H_{\mathrm{CSM}} & = & \sum_{i,j\in N(i)}J_{\left(\sigma_{i},\sigma_{j}\right)}\left(1-\delta_{\sigma_{i},\sigma_{j}}\right)+\sum_{\sigma }\lambda_{\mathrm{Area}}{\left(a_{\sigma }-A_{\sigma }\right)}^{2}\\ & & +\sum_{\sigma }\lambda_{\mathrm{Perimeter}}{\left(p_{\sigma }-P_{\sigma }\right)}^{2} \end{array}$$

where *i* and *j* are neighboring grid sites, *σ* is the cell identity. *N*(*i*) is the Moore neighborhood of grid *i*. The first term of the Hamiltonian sums the energy values, *J*_(_ *_*σ*i,*σ*j_*_)_, between all the neighboring lattice sites *σ_i_* and *σ_j_*_,_ where the term (1 − *δ_*σ*i,*σ*j_*) makes certain that only interactions between different cells are considered in the equation. The second term (the area constraint) and the third term (the perimeter constraint) sum over all cells.

To introduce the adhesive–nonadhesive alternate substrates (FN surface, *S*_FN_, and PEG surface, *S*_PEG_), we employed *H*_substrate_, which is the adhesion energy between cells and substrate. *H*_substrate_ takes the form of

(4) $$H_{\mathrm{substrate}}\left(i\right)=\left\{\begin{array}{cc} \phi_{\mathrm{FN}}, & i\in S_{\mathrm{FN}}\\ \phi_{\mathrm{PEG}}, & i\in S_{\mathrm{PEG}} \end{array}\right.$$

*φ*_FN_ < 0 indicates that FN substrate is conductive for cell adhesion while *φ*_FN_ > 0 means PEG is not suitable for cell adhesion. Then we introduced the activity of actin (*V*_act_) to quality the actin dynamic during cell polarization,^[^ ^31^ ^]^ which was applied to each grid of the cell. *V*_act_ is 0 of the cell at the beginning. When copying to a new grid *j*, if Moore neighborhood of grid *j* contains FN substrate, *V*_act_ in grid *j* is given Act_FN_ (Act_FN_ > 0), or else *V*_act_ is given 0, namely,

(5) $$V_{\mathrm{act}}\left(j\right)=\left\{\begin{array}{cc} {\mathrm{Act}}_{\mathrm{FN}}, & N\left(j\right)\cap S_{\mathrm{FN}}\neq \left\{\right\}\\ 0, & \mathrm{else} \end{array}\right.$$

{} represents empty set. Besides, activity of any grid *k* (*V*_act_(*k*)) will decrease at the end of each Monte Carlo step (MCS) until it reaches zero, namely

(6) $$V_{\mathrm{act}}\left(k\right)=\min\left(V_{\mathrm{act}}\left(k\right)-1,0\right),\;\mathrm{for}\;\mathrm{every}\;\mathrm{MCS}$$

Activity of newborn actin was determined by substrate, which means that FN promotes the polarization of actin while PEG decreases it. The geometric mean value of *V*_act_ of one grid's neighborhood was positively correlated with the “migration energy” given to cells, which increased the tendency of cell movement at this grid. When the cell copies from grid *i* to grid *j*, the energy provided by actin (Δ*H*_act_) is

(7) $$\Delta H_{\mathrm{act}}=\frac{\lambda_{\mathrm{act}}}{{\mathrm{Act}}_{\mathrm{FN}}}\left[\mathrm{GM}(j)-\mathrm{GM}(i)\right]$$

In this equation, GM(*i*) represents the geometric mean of the *V*_act_ of all pixels in *N*(*i*) and belong to the same cell . *λ*_act_ dominant the weight in the energy difference Δ*H* of replication attempts.

Under all rules mentioned above, the probability of cells appearing in the PEG region is very low due to the energy barrier of PEG and the inhibition of actin activity. That is, when confined in FN region, cells can only polarize and move freely in the FN region.

To characterize the positive effect of podosome in migration on PEG, we introduced podosome into the model. Podosome were generated only in the PEG region, which is displayed by a single grid in the model. The generation probability of podosome *P*_grow_ was affected by actin activity. The higher the actin activity value is, the higher the probability will become. The probability of podosome generation in one grid is

(8) $$P_{\mathrm{grow}}\left(i\right)=\left\{\begin{array}{c} \frac{P_{\mathrm{grow}\;0}}{\left(1+\exp\left(-K*\left(V_{\mathrm{act}}\left(i\right)-{\mathrm{Act}}_{\mathrm{threshold}}\right)\right)\right)},\;i\in S_{\mathrm{PEG}}\\ 0,\;\mathrm{else} \end{array}\right.$$

where *P*_grow0_ is the basic probability of podosome generation, Act_threshold_ is the actin activity threshold, *K* is the threshold parameter. The smaller the *K* is, the change of the formation probability is smoother with the change of actin activity. To simulate the turnover of podosome, each generated podosome had a definite lifetime *T*_podo_. There is a probability of *P*_decay_ to decay its lifetime by 1 at the end of each MCS until it reached 0. We denoted *S*_podo_ as the region with a podosome lifetime greater than or equal to 1.

Also, we consider that podosome can anchor and promote actin polarization. Therefore, in the model, the podosome can reduce the blocking energy of PEG during cell replication in its region and increase the actin activity of pixel replication to Act_podo_ (Act_podo_ > Act_FN_). In other words, when the new grid *j* is replicated, its actin activity value rule is changed to

(9) $$V_{\mathrm{act}}\left(j\right)=\left\{\begin{array}{cc} {\mathrm{Act}}_{\mathrm{podo}}, & N\left(j\right)\cap S_{\mathrm{podo}}\neq \left\{\right\}\\ {\mathrm{Act}}_{FN}, & N\left(j\right)\cap S_{\mathrm{podo}}=\left\{\right\}\&N\left(j\right)\cap S_{FN}\neq \left\{\right\}\\ 0, & \mathrm{else} \end{array}\right.$$

The energy of substrate is change to

(10) $$H_{\mathrm{substrate}}\left(j\right)=\left\{\begin{array}{cc} \phi_{\mathrm{FN}}, & j\in S_{\mathrm{FN}}\\ 0, & j\in S_{\mathrm{PEG}}\&N(j)\cap S_{\mathrm{podo}}\neq \left\{\right\}\\ \phi_{\mathrm{PEG}}, & \mathrm{else} \end{array}\right.$$

Therefore, cells can move on the PEG region with the support of podosome, and have higher actin activity than on the FN region, enabling cells to undergo strong polarization on the PEG region.

Next, to simulate the signaling pathway of podosome generation that macrophages need preadhesion on FN substrate to form podosome on PEG region. If the whole part of the cell is located on PEG region, it will detach from FN and shrink into a suspended round shape. Next, we couple the generation probability of podosome and preadhesion on FN region. That is, there is an area threshold of FN, *A*_anchor_. When the total area of cells in FN region *A*_FN_ < *A*_anchor_, additional anchor energy *E*_anchor_ (>0) will be added to prevent cells from detaching from FN. The formula takes the form of

(11) $$H_{\mathrm{anchor}}=\left\{\begin{array}{cc} E_{\mathrm{anchor}}, & 0<A_{\mathrm{FN}}<A_{\mathrm{anchor}}\\ 0, & \mathrm{else} \end{array}\right.$$

If the cell is still unanchored, the generation probability of podosome become 0, so the probability of podosome generation in one grid is changed to

(12) $$P_{\mathrm{grow}}\left(i\right)=\left\{\begin{array}{cc} \frac{P_{\mathrm{grow}\;0}}{\left(1+\exp(-K*\left(V_{\mathrm{act}}\left(i\right)-{\mathrm{Act}}_{\mathrm{threshold}}\right))\right)}, & i\in S_{\mathrm{PEG}}\&A_{\mathrm{FN}}>0\\ 0, & \mathrm{else} \end{array}\right.$$

Due to the high dynamic of the podosome, the podosome will disappear and become “trapped” in the PEG region soon after the generation of it. Also, due to the detachment of the cell, the target area and target perimeter of cells will be reduced.

Finally, we consider that the macrophages movement on the PEG through generating podosome is a process of energy dissipation. Thus, there must be a negative feedback to inhibit continual movement of the cell on PEG region without completing a crossing from one FN region to another. Therefore, we added a feedback mechanisms that when cells reach a new FN region and achieve a anchoring area of *A*_newanchor_, the basic probability of podosome generation on PEG region will reduce to *P*′_grow0_ within a certain time *T*_feedback_, so that cells will complete a crossing instead of staying on the PEG region. After the end of *T*_feedback_, the probability of podosome formation will recover to the original value of *P*_grow0_.

## Conflict of Interest

The authors declare no conflict of interest.

## Author Contributions

L.P. conceived the research and were in charge of the overall direction. L.P. and F.X. designed the experiments. F.X. and P.Z. performed the experiments. F.X. and H.D. performed computational modeling. F.X., J.Y., C.F., M.H., and F.H. analyzed the data. J.Z. and L.C. contributed to the interpretation of the results. F.X. and L.P. wrote the manuscript. L.P. and J.X. supervised the work.

## Supporting information

Supporting Information

Supplemental Movie 1

Supplemental Movie 2

Supplemental Movie 3

Supplemental Movie 4

Supplemental Movie 5

Supplemental Movie 6

Supplemental Movie 7

Supplemental Movie 8

Supplemental Movie 9

Supplemental Movie 10

Supplemental Movie 11

## Data Availability

The data that support the findings of this study are available from the corresponding author upon reasonable request.

## References

[1] J. L. Orgaz , P. Pandya , R. Dalmeida , P. Karagiannis , V. A. Sanchez‐Laorden , J. Albrengues , F. O. Nestle , A. J. Ridley , C. Gaggoli , R. Marais , S. N. Karagiannis , V. Sanz‐Moreno , Nat. Commun. 2014, 5, 4255. 24963846 10.1038/ncomms5255 PMC4118761

[2] S. SenGupta , C. A. Parent , J. E. Bear , Nat. Rev. Mol. Cell Biol. 2021, 22, 529. 33990789 10.1038/s41580-021-00366-6 PMC8663916

[3] A. G. Clark , D. M. Vignjevic , Curr. Opin. Cell Biol. 2015, 36, 13. 26183445 10.1016/j.ceb.2015.06.004

[4] A. Ray , O. Lee , Z. Win , R. M. Edwards , P. W. Alford , D. H. Kim , P. P. Provenzano , Nat. Commun. 2017, 8, 14923. 28401884 10.1038/ncomms14923 PMC5394287

[5] H. Schachtner , S. D. J. Calaminus , S. G. Thomas , L. M. Machesky , Cytoskeleton 2013, 70, 572. 23804547 10.1002/cm.21119

[6] P. R. O'Neill , J. A. Castillo‐Badillo , X. Meshik , V. Kalyanaraman , K. Melgarejo , N. Gautam , Dev. Cell 2018, 46, 9 . 29937389 10.1016/j.devcel.2018.05.029 PMC6048972

[7] Y. J. Liu , M. L. Berre , F. Lautenschlaeger , P. Maiuri , A. Callan‐Jones , M. Heuze , T. Takaki , R. Voituriez , M. Piel , Dev. Cell 2015, 160, 659. 10.1016/j.cell.2015.01.007 25679760

[8] D. S. Eom , D. M. Parichy , Science 2017, 355, 1317. 28209639 10.1126/science.aal2745 PMC5836293

[9] B. Stolp , F. Thelen , X. Ficht , L. M. Altenburger , N. Ruef , V. V. G. Krishna‐Inavalli , P. Germann , N. Page , F. Moalli , A. Raimondi , K. A. Keyser , S. M. S. Jafari , F. Barone , M. S. Dettmer , D. Merkler , M. Iannacone , J. Sharpe , C. Schlapbach , O. T. Fackler , U. V. Nägerl , J. V. Stein , Sci. Immunol. 2020, 5, eaaz4371. 32245888 10.1126/sciimmunol.aaz4371

[10] F. Barros‐Becker , P. Y. Lam , R. Fisher , A. Huttenlocher , J. Cell Sci. 2017, 130, 3801. 28972134 10.1242/jcs.206128 PMC5702045

[11] F. Barros‐Becker , J. M. Squirrell , R. Burke , J. Chini , J. Rindy , A. Karim , K. W. Eliceiri , A. Gibson , A. Huttenlocher , iScience 2020, 23, 101699. 33196024 10.1016/j.isci.2020.101699 PMC7644964

[12] A. J. Davidson , W. Wood , Cell Rep. 2020, 31, 107692. 32460022 10.1016/j.celrep.2020.107692 PMC7262594

[13] R. Sridharan , B. Cavanagh , A. R. Cameron , D. J. Kelly , F. J. O'Brien , Acta Biomater. 2019, 89, 47. 30826478 10.1016/j.actbio.2019.02.048

[14] F. Y. McWhorter , T. Wang , P. Nguyen , W. F. Liu , Proc. Natl Acad. Sci. U. S. A. 2013, 110, 17253. 24101477 10.1073/pnas.1308887110 PMC3808615

[15] K. M. Hansson , S. Tosatti , L. Isaksson , J. Wettero , M. Textor , T. L. Lindahl , P. Tengvall , Biomaterials 2005, 26, 861. 15353197 10.1016/j.biomaterials.2004.03.036

[16] J. Bluemmel , N. Perschmann , D. Aydin , J. Drinjakovic , T. Surrey , M. Lopez‐Garcia , H. Kessler , J. P. Spatz , Biomaterials 2007, 28, 4739. 17697710 10.1016/j.biomaterials.2007.07.038

[17] J. E. Bear , J. M. Haugh , Curr. Opin. Cell Biol. 2014, 30, 74. 24999834 10.1016/j.ceb.2014.06.005 PMC4177959

[18] D. L. Bodor , W. Pönisch , R. G. Endres , E. K. Paluch , Dev. Cell 2020, 52, 550. 32155438 10.1016/j.devcel.2020.02.013

[19] J. Renkawitz , M. Sixt , EMBO Rep. 2010, 11, 744. 20865016 10.1038/embor.2010.147 PMC2948197

[20] J. C. Herron , S. Hu , T. Watanabe , A. T. Nogueira , B. Liu , M. E. Kern , J. Aaron , A. Taylor , M. Pablo , T. L. Chew , T. C. Elston , K. M. Hahn , Nat. Commun. 2022, 13, 4363. 35896550 10.1038/s41467-022-32038-0 PMC9329332

[21] F. Hu , D. Zhu , H. Dong , P. Zhang , F. Xing , W. Li , R. Yan , J. Zhou , K. Xu , L. Pan , J. Xu , iScience 2022, 105514. 36425766 10.1016/j.isci.2022.105514 PMC9678738

[22] B. Huang , X. Wang , M. Bates , X. Zhuang , Science 2008, 319, 810. 18174397 10.1126/science.1153529 PMC2633023

[23] S. Tsuboi , J. Immunol. 2007, 178, 2987. 17312144 10.4049/jimmunol.178.5.2987 PMC1855218

[24] A. Olivier , L. Jeanson‐Leh , G. Bouma , D. Compagno , J. Blondeau , K. Seye , S. Charrier , S. Burns , A. J. Thrasher , O. Danos , W. Vainchenker , A. Galy , Mol. Ther. 2006, 13, 729. 16360341 10.1016/j.ymthe.2005.11.003

[25] K. Clark , M. Langeslag , B. van Leeuwen , L. Ran , A. G. Ryazanov , C. G. Figdor , W. H. Moolenaar , K. Jalink , F. N van Leeuwen , EMBO J. 2007, 25, 290. 10.1038/sj.emboj.7600931 PMC1383514 16407977

[26] H. Schachtner , S. D. J. Calaminus , A. Sinclair , J. Monypenny , M. P. Blindell , C. Leon , T. L. Holyoake , A. J. Thrasher , A. M. Michie , M. Vukovic , C. Gachet , G. E. Jones , S. G. Thomas , S. P. Watson , L. M. Machesky , Blood 2013, 121, 2542. 23305739 10.1182/blood-2012-07-443457

[27] H. Tanaka , H. H. Wang , S. E. Thatcher , H. Hagiwara , H. Takano‐Ohmuro , K. Kohama , J Pharmacol Sci 2015, 128, 78. 25986486 10.1016/j.jphs.2015.03.002

[28] S. F. G. Van Helden , M. M. Oud , B. Joosten , N. Paterse , C. G. Figdor , F. N. van Leeuwem , J. Cell Sci. 2008, 121, 1096. 18334555 10.1242/jcs.020289

[29] F. Graner , J. A. Glazier , Phys. Rev. Lett. 1992, 69, 2013. 10046374 10.1103/PhysRevLett.69.2013

[30] I. Niculescu , J. Textor , R. J. De Boer , PLoS Comput. Biol. 2015, 11, e1004280. 26488304 10.1371/journal.pcbi.1004280 PMC4619082

[31] I. M. N. Wortel , I. Niclescu , P. M. Kolijn , N. S. Gov , R. J. de Boer , L. Textor , Biophys. J. 2021, 120, 2609. 34022237 10.1016/j.bpj.2021.04.036 PMC8390880

[32] M. Bergert , A. Erzberger , R. A. Desai , I. M. Aspalter , A. C. Oates , G. Charras , G. Salbreux , E. K. Paluch , Nat. Cell Biol. 2015, 17, 524. 25774834 10.1038/ncb3134 PMC6485532

[33] S. J. Sofia , V. Premnath , E. W. Merrill , Macromolecules 1998, 31, 5059. 9680446 10.1021/ma971016l

[34] L. Aoun , A. Farutin , N. Garcia‐Seyda , P. Negre , M. S. Rizvi , S. Tlili , S. Song , X. Luo , M. Biarnes‐Pelicot , R. Galland , J.‐B. Sibarita , A. Michelot , C. Hivroz , S. Rafai , M.‐P. Valignat , C. Misbah , O. Theodoly , Biophys. J. 2020, 119, 1157. 32882187 10.1016/j.bpj.2020.07.033 PMC7499394

[35] N. P. Barry , M. S. Bretscher , Proc. Natl Acad. Sci. U. S. A. 2010, 107, 11376. 20534502 10.1073/pnas.1006327107 PMC2895083

[36] P. Friedl , B. Weigelin , Nat. Immunol. 2008, 9, 960. 18711433 10.1038/ni.f.212

[37] S. L. Vecchio , R. Thiagarajan , D. Caballero , V. Vigon , L. Navoret , R. Voituriez , Cell Syst. 2020, 10, 535 . 32553185 10.1016/j.cels.2020.05.005

[38] D. Garbett , A. Bisaria , C. Yang , D. G. McCarthy , A. Hayer , W. E. Moerner , T. M. Svitkina , T. Meyer , Nat. Commun. 2020, 11, 4818. 32968060 10.1038/s41467-020-18586-3 PMC7511357

[39] M. Krause , K. Wolf , Cell Adhes. Migr. 2019, 9, 357. 10.1080/19336918.2015.1061173 PMC4955366 26301444

[40] K. M. Yamada , M. Sixt , Nat. Rev. Mol. Cell Biol. 2019, 20, 738. 31582855 10.1038/s41580-019-0172-9

[41] C. Yu , N. B. M. Rafiq , A. Krishnasamy , K. L. Hartman , G. E. Jones , A. D. Bershadsky , M. P. Sheetz , Cell Rep. 2013, 5, 1456. 24290759 10.1016/j.celrep.2013.10.040 PMC3898747

[42] Y. Li , Z. Ren , Y. Wang , Y. Z. Dang , B. X. Meng , J. Zhang , J. Wu , N. Wen , Exp. Cell Res. 2018, 370, 373. 29966664 10.1016/j.yexcr.2018.06.039

[43] Y. Zhou , Z. Feng , F. Cao , X. Liu , X. Xia , C. Yu , J. Cell Sci. 2020, 133, jcs234385. 32393599 10.1242/jcs.234385

[44] S. Campillo , L. Bohorquez , E. Gutiérrez‐Calabrés , D. García‐Ayuso , V. Miguel , M. Griera , Y. Calle , S. Frutos , M. Rodríguez‐Puyol , D. Rodríguez‐Puyol , L. Calleros , Exp. Mol. Med. 2022, 54, 226. 35246616 10.1038/s12276-022-00738-8 PMC8980039

[45] K. Pal , Y. Zhao , Y. Wang , X. Wang , J. Cell Biol. 2019, 220, e202008079.

[46] A. Labernadie , C. Thibault , C. Vieu , I. Maridonneau‐Parini , G. M. Charrière , Proc. Natl Acad. Sci. U. S. A. 2010, 107, 21016. 21081699 10.1073/pnas.1007835107 PMC3000246

[47] N. B. M. Rafiq , Y. Nishimura , S. V. Plotnikov , V. Thiagarajan , Z. Zhang , S. Shi , M. Natarajan , V. Viasnoff , P. Kanchanawong , G. E. Jones , A. D. Bershadsky , Nat. Mater. 2019, 18, 638. 31114072 10.1038/s41563-019-0371-y

[48] F. Xing , P. Zhang , P. Jiang , Z. Chen , J. Yang , F. Hu , I. Drevensek‐Olenik , Z. Zhang , L. Pan , J. Xu , ACS Appl. Mater. Interfaces 2018, 10, 2937. 29283550 10.1021/acsami.7b15759

