# Supplementary material for: Mesenchymal Migration on Adhesive–Nonadhesive Alternate Surfaces in Macrophages
Source: Adv Sci (Weinh). 2023 May 21;10(23):2301337. doi: 10.1002/advs.202301337 (PMC10427406; doi:10.1002/advs.202301337)
Supplement: Supplementary file 1 — Supporting Information [file ADVS-10-2301337-s003.pdf]

ADVANCED SCIENCE
Open Access

Supporting Information

for *Adv. Sci.*, DOI 10.1002/advs.202301337

Mesenchymal Migration on Adhesive–Nonadhesive Alternate Surfaces in Macrophages

Fulin Xing, Hao Dong, Jianyu Yang, Chunhui Fan, Mengdi Hou, Ping Zhang, Fen Hu, Jun Zhou, Liangyi Chen, Leiting Pan and Jingjun Xu*

Supporting Information

Mesenchymal migration on alternate non-adhesive surfaces in macrophages

Fulin Xing, Hao Dong, Jianyu Yang, Chunhui Fan, Mengdi Hou, Ping Zhang, Fen Hu, Jun Zhou, Liangyi Chen, Leiting Pan, Jingjun Xu

Correspondence to: plt@nankai.edu.cn

This file includes:

Figures. S1 to S7
Captions for Movies M1 to M11

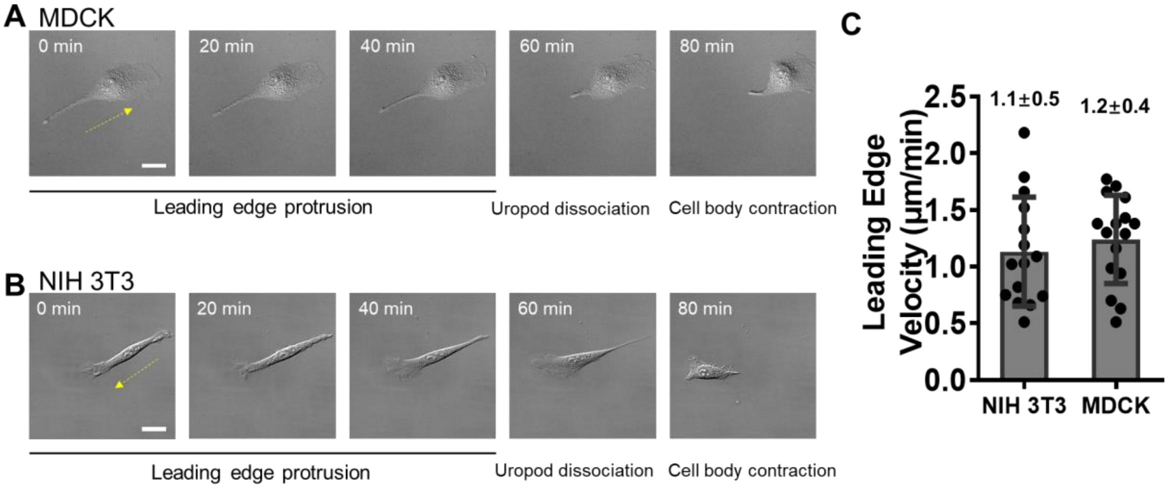

Figure S1. Mesenchymal migration of MDCK and NIH 3T3 cells on uniform substrates.
A,B) Typical mesenchymal migration of MDCK and NIH 3T3 cells. Scale bars, 20 μm. **C)** Statistical velocities of the leading edge in MDCK and NIH 3T3 cells.

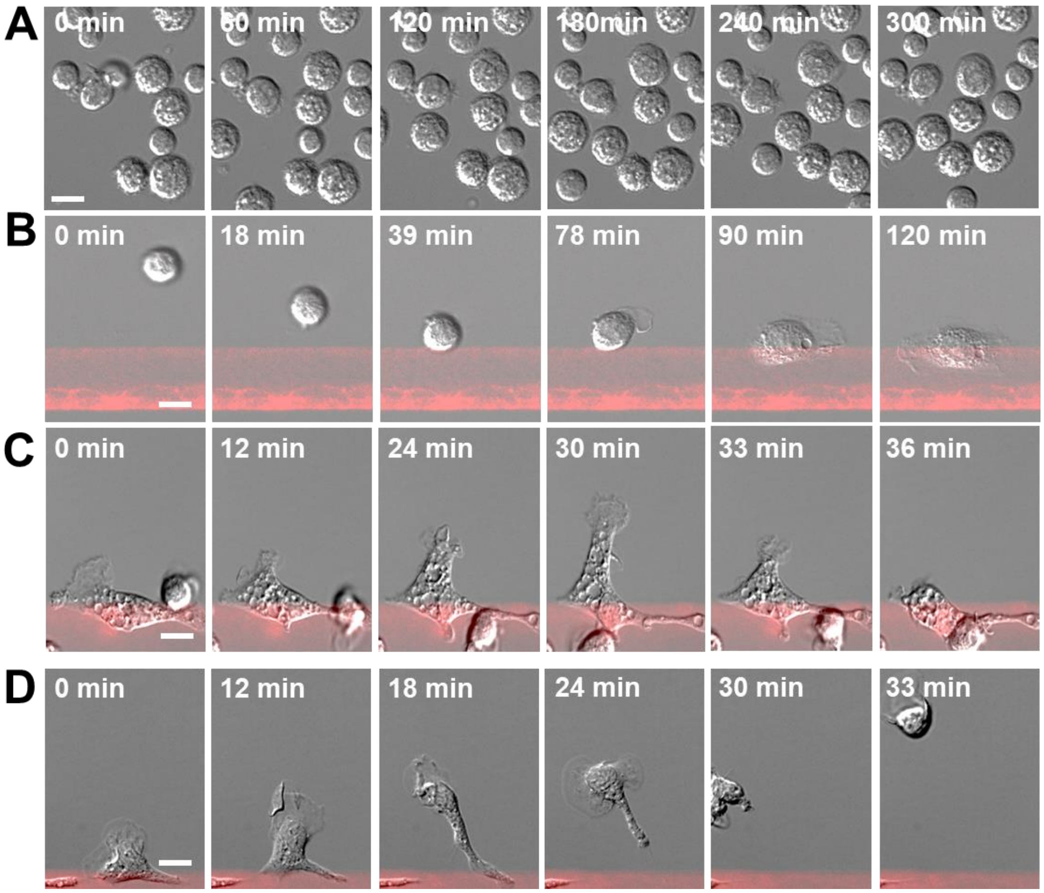

Figure S2. Mesenchymal migration on the adhesive-nonadhesive alternate surface needs pre-adhesive on the FN region.

A) Cells keep a suspending round morphology on the pure PEG region. **B)** Macrophage adheres and spreads when it reaches a FN region. **C)** The cell extends out to PEG region and then retracts. **D)** Macrophage extends out to the PEG region and then detaches from the FN region followed by turning round. Scale bars, 10 μm from A) to D).

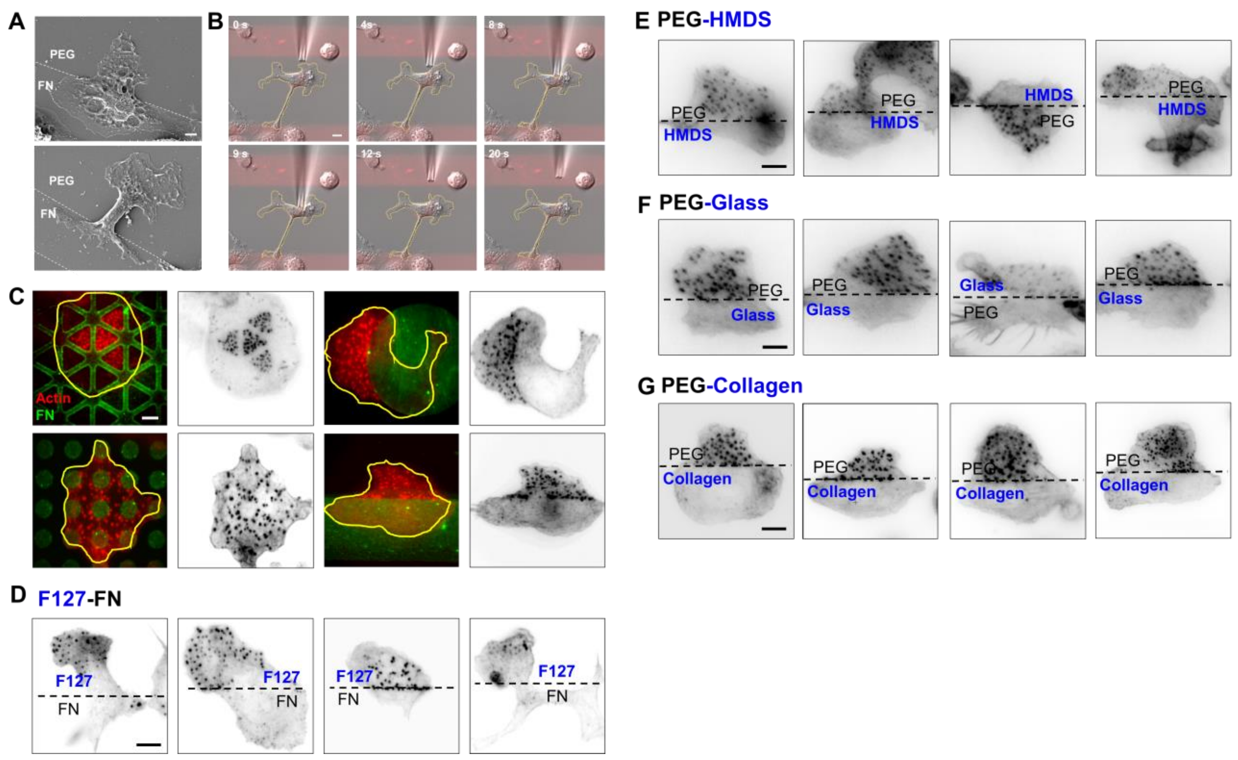

Figure S3. Macrophages adhere and form podosomes on non-adhesive regions.

A) Scanning electron microscopy images of macrophages on FN-PEG boundary of the alternate non-adhesive surface. Scale bar, 2 µm. **B**) Gentle mechanical stimulation could not move the cell extending on the PEG region. Scale bar, 5µm. **C**) Cells form podosomes on the PEG region in various patterns, including meshwork patterns, circle arrays, circular patterns, and stripe patterns. Scale bars, 5µm. **D**) Macrophages could extend to the F127 (another anti-adhesion regent) region and form podosomes. Scale bars, 10 µm. **E,F,G**) Macrophages could extend to the PEG region and form podosomes on alternate PEG/HMDS E), PEG/glass F), and PEG/collagen G) substrates. Scale bars, 10 µm.

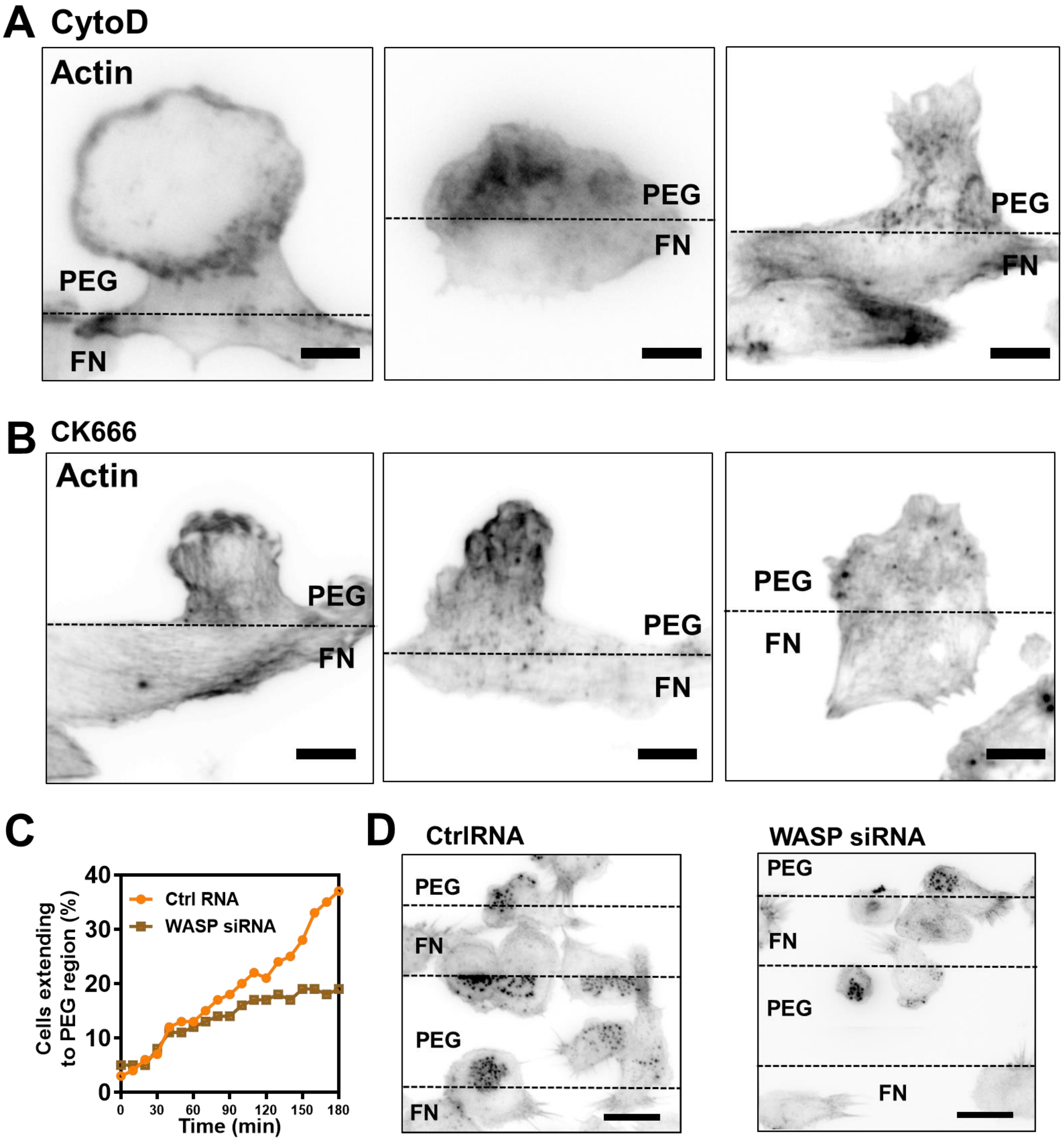

Figure S4. Drugs and siRNA disassemble the podosome on PEG regions and block cell motility on the adhesive-nonadhesive alternate surfaces.

A,B) CytoD (5 μM) A) and CK666 (20 μM) B) could block the formation of podosomes. Scale bars, 10 μm. **C**) WASP siRNA reduced the macrophage motility on adhesive-nonadhesive alternate surfaces. **D**) WASP siRNA decreased the formation of podosomes in macrophage on adhesive-nonadhesive alternate surfaces. Scale bars, 10 μm.

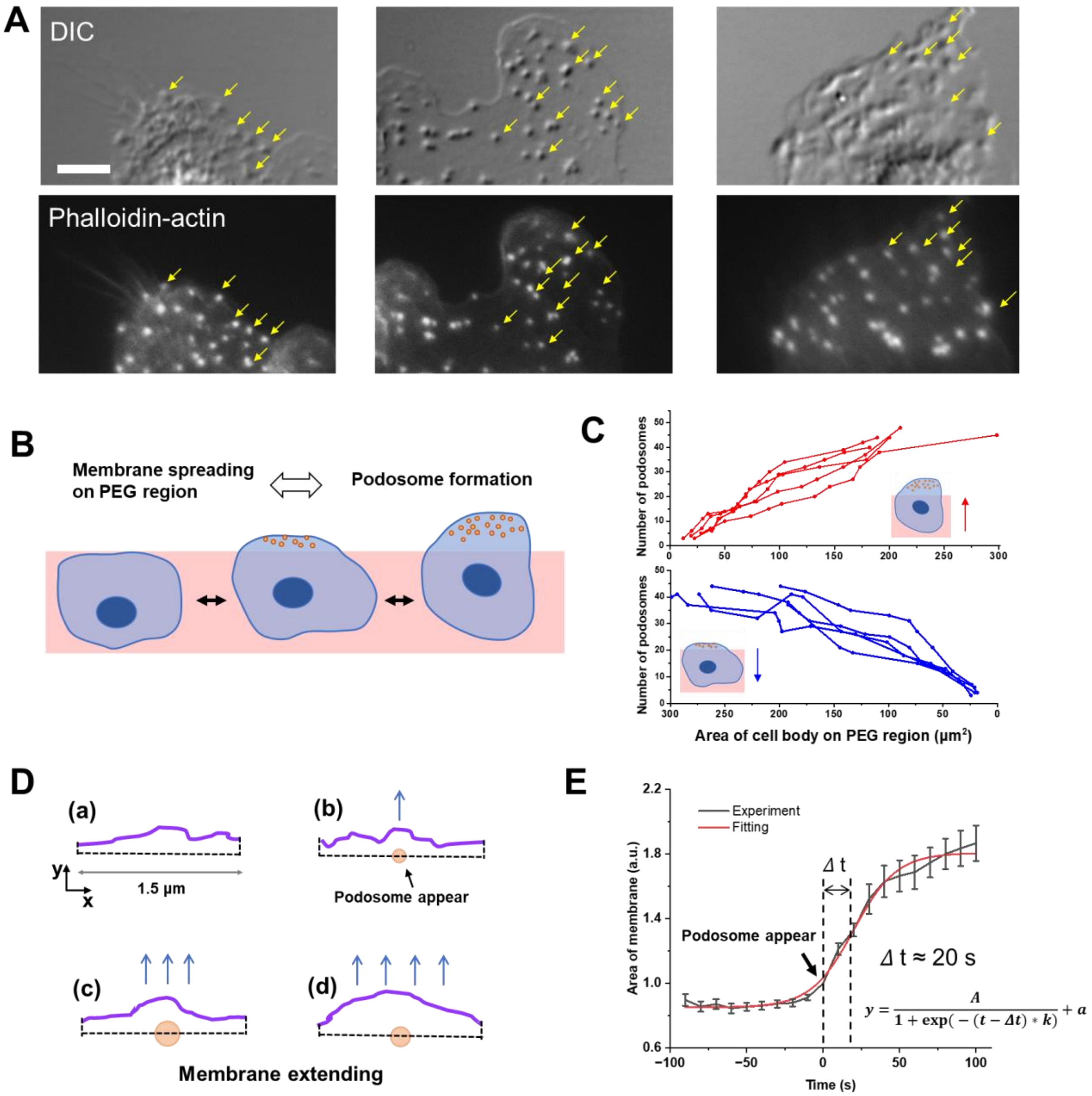

Figure S5. Podosome dynamic and membrane protrusion by DIC imaging.

A) Protuberances in DIC images match podosomes labeled by phalloidin. Scale bar, 5 μm. **B)** Schematic diagram of cell spreading and podosome formation on PEG region. **C)** The area of cell body on the PEG region is positively associated with the number of podosomes on PEG region. Upper: cells protruding out to PEG region. Lower: cells retracting back to FN region. N=5, different lines indicate different cells. **D)** Membrane protrusion analysis. (a) Membrane maintains stable before podosome formation. (b) A single podosome appears. The position of the podosome center was set as the central point of the 1.5-μm dashed line. (c) Membrane protrudes out after the formation of podosome. (c) Neighboring membrane protrudes out. The area of the enclosed region containing the 1.5-μm length dashed line and the outline of the leading-edge

membrane (violet curves) was measured every 10 s in 200 s duration prior and after the podosome appearance. **E)** The change of the area prior and after the podosome appearance. The area at each time point was normalized by the area at t=0. Data was obtained from 6 cells in 3 independent experiments and fitted by logistic function (red curve). The time lag from t=0 to the point when the membrane protrudes fastest was $\Delta t \approx 20$ s according to the fitting. a.u., arbitrary unit.

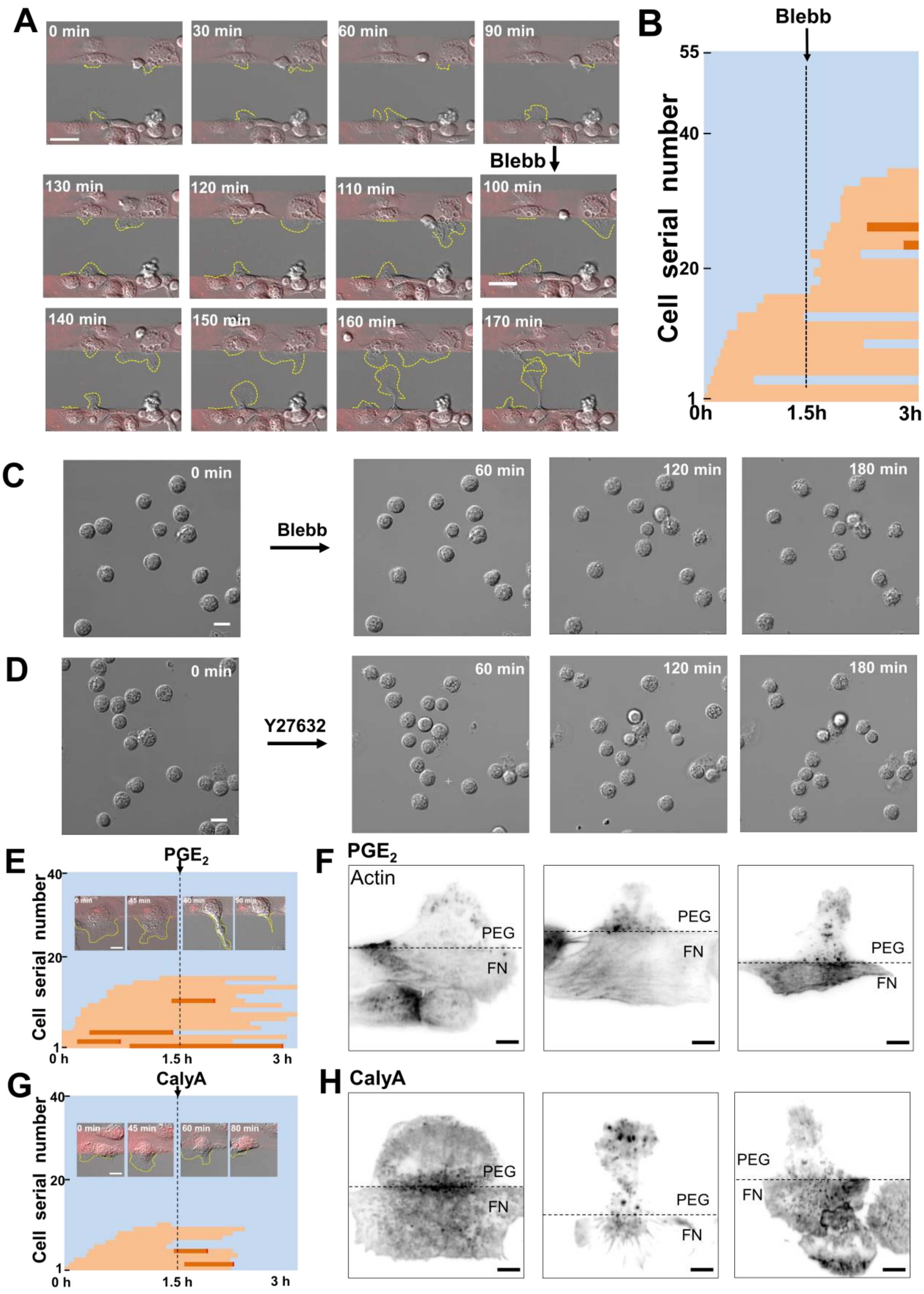
A
0 min
30 min
60 min
90 min
Blebb
130 min
120 min
110 min
100 min
140 min
150 min
160 min
170 min
B
Blebb
Cell serial number
55
40
20
1
0h
1.5h
3h
C
0 min
Blebb
60 min
120 min
180 min
D
0 min
Y27632
60 min
120 min
180 min
E
PGE$_2$
Cell serial number
40
20
1
0 h
1.5 h
3 h
F
PGE$_2$
Actin
PEG
FN
G
CalyA
Cell serial number
40
20
1
0 h
1.5 h
3 h
H
CalyA
PEG
FN

Figure S6. Inhibition of myosin IIA facilitate cell motility on the adhesive-nonadhesive alternate surfaces while activation of myosin pathway inhibited podosome formation and migration on the adhesive-nonadhesive alternate surfaces.

A,B) Blebb promotes cell motility on the alternate non-adhesive PEG surface. Scale bar, 20 μm. **C,D**) Macrophages could not adhere to pure PEG surfaces treatment with Blebb C) and Y27632 D) treatment. Scale bars, 10 μm. **E,F,G,H**) PGE_2 E) and CalyA G) decrease cell motility on the alternate non-adhesive PEG surface. Scale bars, 10 μm. 25 μM PGE_2 F) and 5 nM CalyA H) could block the formation of podosomes. Scale bars, 5 μm.

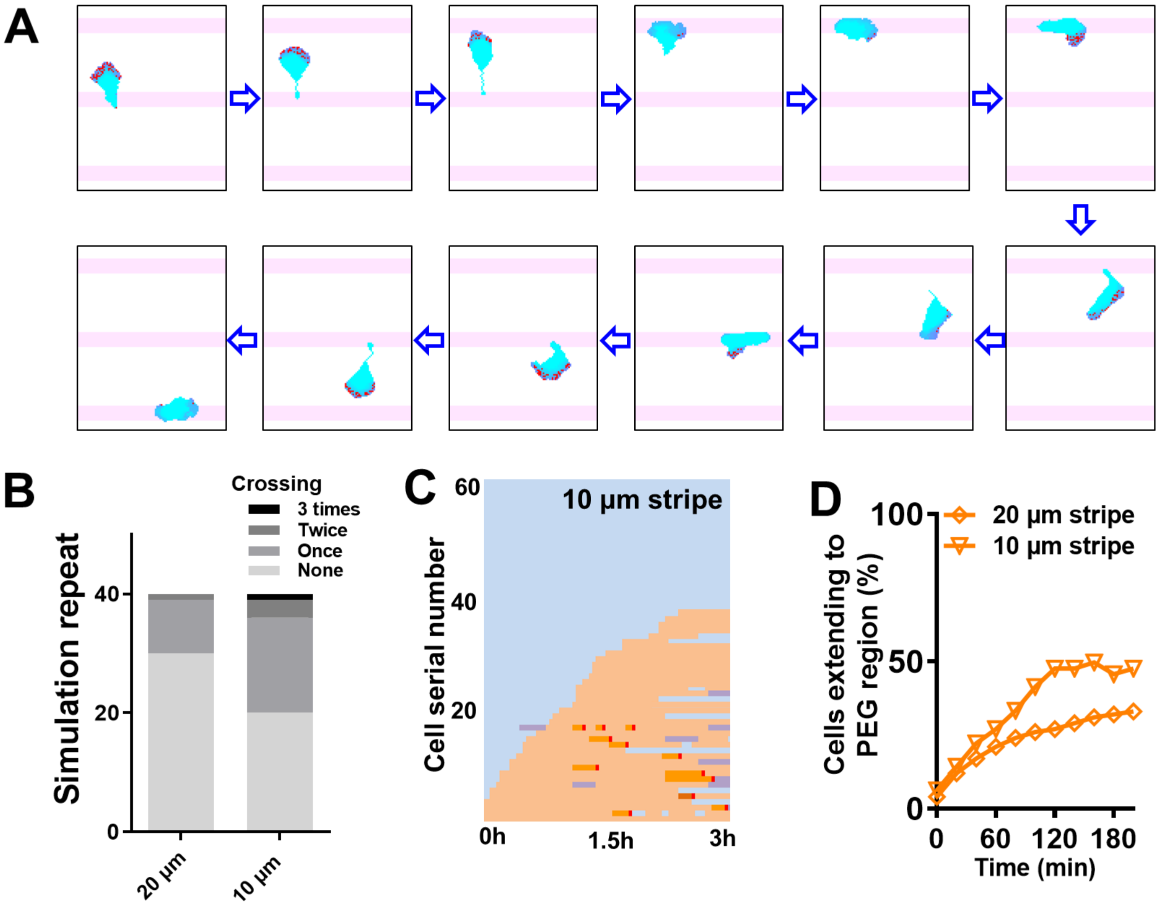

Figure S7. Narrower FN stripes promote cell migration across non-adhesive gaps.

A) Simulation presentation of cell migration on the alternate non-adhesive surface with 10 μm FN stripe. **B)** Number of crossings in 40 repeats of simulation in 20 μm FN stripe and 10 μm FN stripe groups. **C)** Migration diagram of 10 μm FN stripe group from experimental results of movie S10. Cells could extend to the PEG region and complete crossings on the 10 μm FN stripe more easily than cells on the 20 μm FN stripe. **D)** Fraction of cells extending to the PEG region over time in the 20 μm and 10 μm FN stripe groups.

Movie M1. Mesenchymal migration can happen on the adhesive-nonadhesive alternate surfaces in macrophages.

Part I shows the time-lapse of a macrophage migrating from one FN region to another non-adhesive PEG region, related to Fig. 1A and B. Part II shows the migration of macrophages on stripe patterns with PEG gaps of 40 μm and 80 μm.

Movie M2. Motility of other cells on the adhesive-nonadhesive alternate surfaces

Part I shows that selected cancer cells can not extend to the PEG region. Part II shows that selected cell lines cannot extend to the PEG region. Part III shows that selected primary cells cannot migrate across the PEG region (human neutrophils can extend to the PEG region but cannot elongate and reach another FN region). Part IV shows that rat macrophages and raw264.7 cells can migrate across the PEG region, and that mouse macrophages can migrate across the F127-FN and PEG-collagen region.

Movie M3. Mesenchymal migration on adhesive-nonadhesive alternate surfaces needs pre-adhesion on the FN region.

Part I shows that cells cannot adhere on pure PEG until they reach an FN region. Part II shows that cells extending to the PEG region will retract or detach when failing to reach another FN region.

Movie M4. Gentle mechanical stimulation cannot move the cell extending on the PEG region.

This Movie M shows that macrophage adheres on the PEG surface.

Movie M5. Podosome dynamic during migration on the adhesive-nonadhesive alternate surfaces.

Time-lapse of macrophage labeled by SiR-actin using TIRFM. Part I shows that podosomes appear when extending to the PEG region. Part II shows that the podosomes disappear when this part reaches the FN region. Part III shows that the podosomes disappear before retraction.

Movie M6. Cells retract to the FN region upon CytoD and CK666 treatment. This Movie M shows that podosomes are essential for cell migration on the alternate non-adhesive substrates.

Movie M7. Macrophages motility was reduced by WASP siRNA. Less cells extended to PEG region on the alternate non-adhesive substrates upon WASP siRNA transfection.

Movie M8. Podosome turnover in DIC images.

The left panel shows that membrane protuberances keep turnover on the PEG region. The right panel shows that the membrane protrudes out after an adjacent podosome appears.

Movie M9. Mesenchymal migration on the adhesive-nonadhesive alternate surfaces is enhanced upon Blebb and Y27632 treatment and inhibited upon PGE_2 and CalyA. Inhibition of myosin enhanced cell motility (part I) while activation of myosin IIA decreased it (part III). Inhibition of myosin IIA promotes this motility on adhesive-nonadhesive alternate surface, but does not lead to cell adhesion on pure PEG surface (part II).

Movie M10. Simulation of mesenchymal migration on the adhesive-nonadhesive alternate surfaces.

Part I shows a cell crossing the PEG gap. Part II shows the cell detaches and re-adheres on the FN region. Part III shows the cell motility is enhanced when the generation of podosomes is up-regulated. Part IV simulates that the cell migrates on triangular lattice circular patterns. MCSs, Monte Carlo steps.

Movie M11. Cell migration on narrow stripe patterns.

Part I shows the simulation result of cell migrating on stripe pattern with 10 μm FN stripe and 40 μm PEG gap. Part II shows the experimental result of cells migrating on the same stripe pattern. MCSs, Monte Carlo steps.
